# Peratrial Device Closure of Atrial Septal Defect Under Transesophageal Echocardiographic Guidance without Fluoroscopy Compared to Conventional On-Pump Surgical Closure

**DOI:** 10.21470/1678-9741-2016-0021

**Published:** 2017

**Authors:** Zhixiang Guo, Chengxin Zhang, Huan Wang, Shenglin Ge

**Affiliations:** 1 Cardiovascular Surgery Department, The First Affiliated Hospital of Anhui Medical University, Hefei, Anhui, China.; 2 Department of Anaesthesia, The First Affiliated Hospital of Anhui Medical University, Hefei, Anhui, China.

**Keywords:** Heart Defects, Congenital, Transesophageal Echocardiography, Minimally Invasive Surgical Procedures

## Abstract

**Objective:**

This study is designed to evaluate the advantages between peratrial device closure under transesophageal echocardiographic guidance and open heart surgery in atrial septal defect.

**Methods:**

From November 2011 to September 2014, 28 patients with atrial septal defect were treated. Fourteen patients received peratrial device closure under transesophageal echocardiographic guidance (TEE group) and 14 patients received cardiopulmonary bypass (CPB group). Clinical parameters during intraoperative and postoperative periods were examined.

**Results:**

All patients recovered after surgery without serious complications. Compared with that in CPB group, clinical observations in TEE group showed significant decreases in the operation time (193.6±35.5 *vs.* 77.4±22.7 min, *P*<0.05), periods in intensive care unit (31.6±23.3 *vs.* 17.5±8.1 hours, *P*<0.05), fluid volume after operation (502.5±439.3 *vs*. 32.5±7.3 ml, *P*<0.05), postoperative length of hospital stay (8.9±2.8 *vs*. 6.8±2.4 days, *P*<0.05) and total hospitalization cost (7205.9±1617.6 *vs.* 5882.3±441.2 $, *P*<0.05).

**Conclusion:**

The peratrial device closure of atrial septal defect under transesophageal echocardiographic guidance is a mini-invasive, simple, safe and effective intervention. Its use in the clinical practice should be encouraged.

**Table:** 

Abbreviations, acronyms & symbols	
ASD CHD CPB ICU PET TEE VSD	= Atrial septal defect = Congenital heart disease = Cardiopulmonary bypass = Intensive care unit = Polyethylene terephthalate = Transesophageal echocardiography = Ventricular septal defect

## INTRODUCTION

Congenital heart disease (CHD) is the most common congenital malformation with the incidence rate of about 0.6% to 1% of all new births^[1,2]^. Ventricular septal defect (VSD) and atrial septal defect (ASD) are two kinds of CHD with about 30% of the total number of CHD in China^[3]^. Currently, the main treatment methods for CHD include cardiopulmonary bypass (CPB) surgery, percutaneous catheter interventional occlusion and recently developed transesophageal echocardiography (TEE) guided percutaneously with minimally invasive incision closure. Although surgical repair has been proven to be safe and effective in conventional CPB, there are some complications related to surgery or CPB^[4]^. In recent years, the method of transcatheter occlusion has achieved good curative effect, but it is limited by the age and weight of the patient, and will cause a certain degree of radiation injury. Therefore, the small incision closure with TEE guided with minimally invasive technique has attracted the attention of many surgeons^[5,6]^. In the present study, we compared the clinical outcomes of routine surgical procedures in the treatment of CHD with the closure of ASD under the guidance of TEE.

## METHODS

### Patients

From 2011 to 2014, 14 patients (3 males and 11 females) with ASD underwent minimally invasive transthoracic occlusion surgery under guidance of TEE (TEE group). Median age was 16 years (range, 2 to 46 years). Body mass ranged from 11.5 to 72 kg (Table 1). Mild, moderate and severe pulmonary hypertension were presented for 5, 2 and none patients, respectively. Fourteen cases (6 males and 8 females) with ASD undergoing CPB) surgery at the same period were used as control (CPB group). Median age was 21 years (range, 3 to 55 years). Body mass ranged from 12 to 76 kg. Mild, moderate and severe pulmonary hypertension were presented for 5, 2 and 1 patients, respectively. The maximum and minimum diameters for ASD were 25 mm and 5 mm, respectively. Based on the World Health Organization definition, mild, moderate and severe pulmonary hypertension refer to the pulmonary artery systolic pressure of 30~40 mmHg, 40~70 mmHg and >70 mmHg, respectively.

**Table 1:** Age, weight and gender in two groups.

	TEE group	CPB group	t	*P* value
Age (years)	18.3	24.9	1.15	0.26
Weight (kg)	37.7	46.8	1.25	0.22
Gender (% of male)	20	46	1.05 (chisq)	0.30

This study was a prospective and non-randomized study and it was approved by the hospital medical ethics committee. All patients were informed about the procedures and complications, and voluntarily signed the consent form.

### The Inclusion Criteria for ASD

The inclusion criteria for ASD patients in both groups are: (1) age >1-year-old and weight > 8 kg; (2) ASD diameter form 5 mm to 34 mm; (3) the distances from edge of the defect to the coronary sinus, the superior and inferior vena cava and the pulmonary vein opening > 5 mm respectively, to the atrioventricular valve > 7 mm; (4) the diameter of atrial septum > diameter of left atrial side plate of the occluder; (5) no other cardiac malformations required surgery.

### Materials and Procedures

**Fig. 1:**
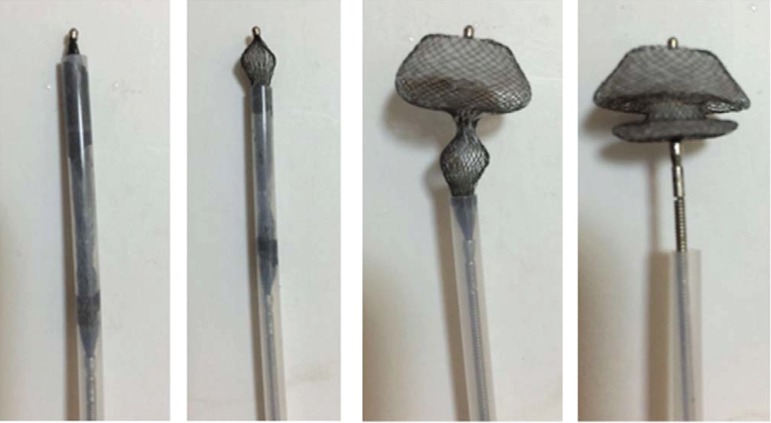
Appearance and structure of Lifetech ASD Occluder.

ASD occluders were provided by Lifetech Scientific (Shenzhen Co., China) (Figure 1). The ASD occluder is covered by a polyethylene terephthalate (PET) membrane that minimizes the chance of clot formation and has a small volume to get into lower profile sheath. ASD occluders have safe long-term biocompatibility and promote the growth of endothelial tissue, lessen thrombus complication and effectively reduce atrioventricular block occurrence.

**Fig. 2:**
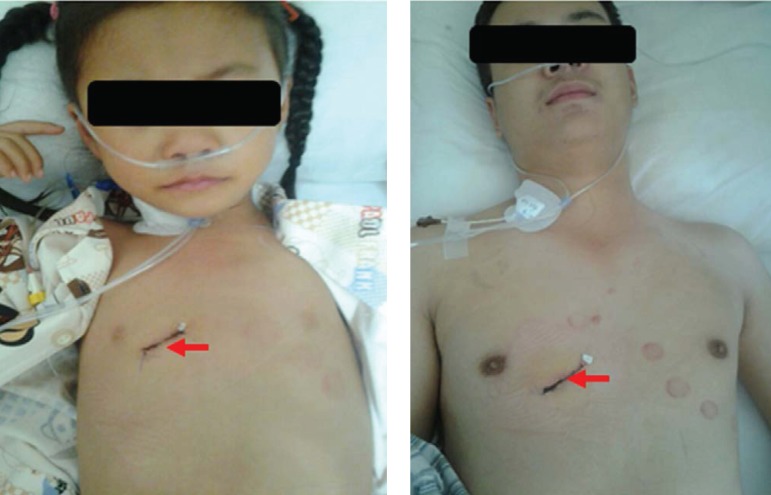
Surgical incision in a child (left) and an adult (right), both shown with a red arrow.

The patients were placed in the supine position followed by inhaled general anesthesia. The TEE checks were used to verify the defect malformations. The surgical procedures were described previously^[7]^. Briefly, patients were placed on the operation-table at prone position. The incisions on the chest wall at the fourth intercostal space right lateral sternal for ASD were 2.0 ~ 3.0 cm in length (Figure 2). Heparin with dose of 200 U/kg was intravenously injected after pericardium incision. At the site of the selected cardiac wall, double U-shape suture were sewed followed by placing the outer self-made delivery sheath and guide probe into the cardiac chamber or main pulmonaryartery. The delivery rod was pushed into the corresponding chamber of the heart along the guide wire through the defects under TEE surveillance. The guide wire was pulled out while the occluder was placed into the inner delivery sheath. The “push-pull” test was done to adjust the position of the occluder release. The delivery sheath and the safe wire were cut off and pulled out of the heart, then the double U-shape suture were ligated strictly after lungs inflation. The thoracic incisions were closed layer by layer.

Patients in CPB group underwent conventional open surgery. Briefly, the incision was performed on median chest and conventional extracorporeal circulation was established. Under 28-32ºC of hypothermia, the ascending aorta was blocked. Atrial septal defect repair surgery was done within 20-55 minutes during the cardiac arrest.

### Statistical Methods

R 3.1.2 software was used to analyze the data showing by mean ± standard deviation. Two groups were compared using independent samples t test, *P*<0.05 for statistical differences.

## RESULTS

**Fig. 3:**
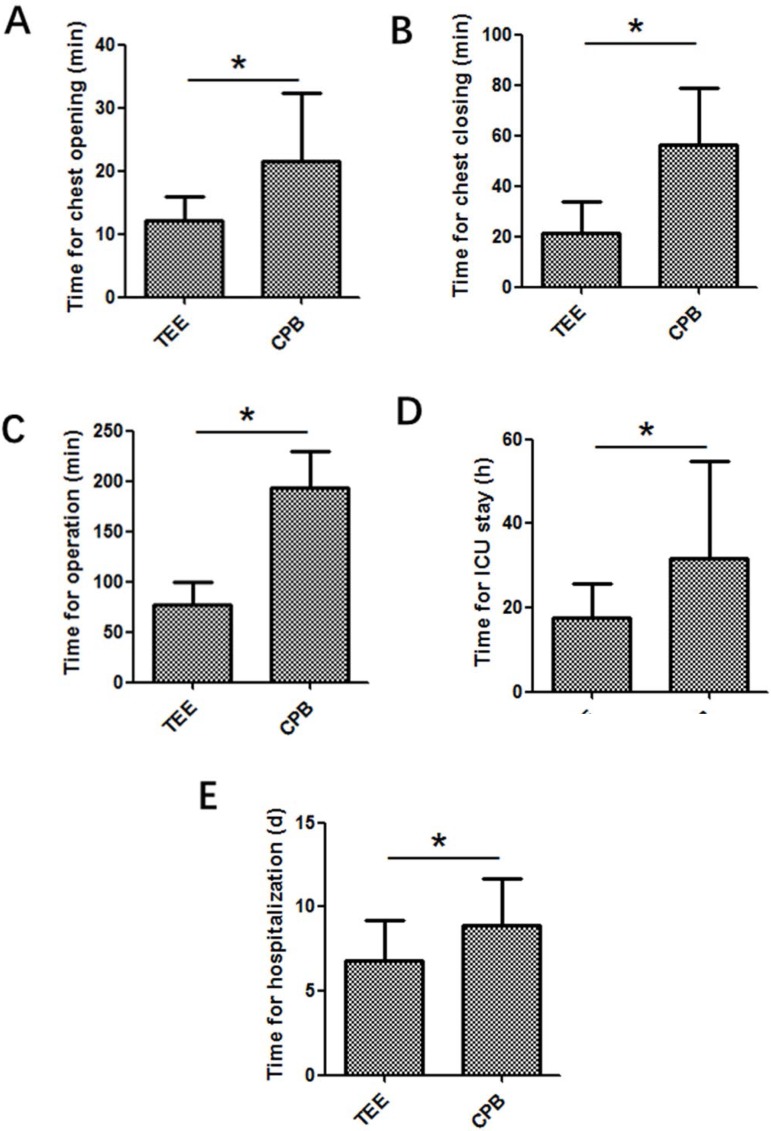
Time for chest opening (A), time for chest closing (B), time for operation (C), time for ICU stay (D) and time for hospitalization (E).

**Fig. 4:**
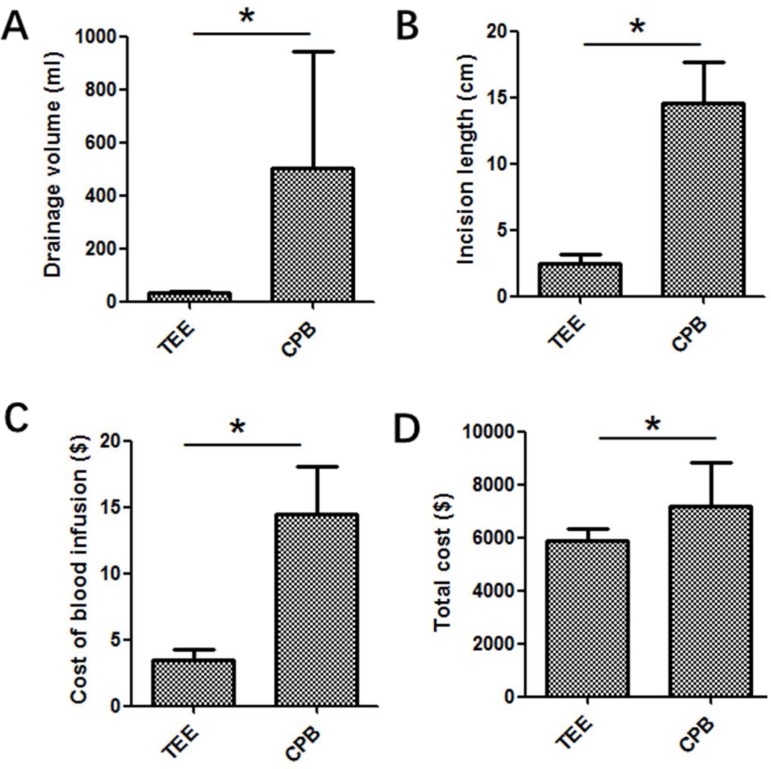
Volume of drainage (A), length of incision (B), cost of blood infusion (C) and full cost (D).

The defect closure or repair in both groups was successfully completed, and all patients recovered after operation without severe complications. Compared with CPB group, TEE group showed significant decreases in time for chest opening (Figure 3A), chest closing (Figure 3B), time for operation (Figure 3C), intensive care unit (ICU) stay (Figure 3D), length of stay (Figure 3E), volume of drainage (Figure 4A), length of incision (Figure 4B), cost of blood infusion (Figure 4C) and full cost (Figure 2D). Six point sixty seven percent of patients in TEE group and 53.33% in CPB group had a small amount of pleural effusion after surgery. Six point sixty seven percent of patients in TEE group and 86.67% in CPB group got perioperative infusion of blood products. There were no serious complications, no second chest opening to stop bleeding and no pericardial effusion during the period of hospitalization in the two groups. During the 12 to 24 months of the routine follow-up, no death, no residual shunt and no serious arrhythmia were found.

## DISCUSSION

CPB routine surgery is the main approach for the treatment of CHD^[1,8]^. However, CPB had disadvantages such as damage of the sternum structure and surrounding tissue caused by sternotomy and retractor exposure of unsuccessful surgical incision and slow recovery of postoperative period and prolonged hospitalization. In this regard, the cardiovascular surgeon has been exploring a less invasive surgical approach, including transcatheter closure technique or heart defect repair with video assisted thoracoscopic surgery^[9]^.

Since King et al.^[10]^ reported the first case of the catheter based closure of VSD in 1976, transcatheter closure techniques have been rapidly developed with the successful development of the Amplatzer occluder and other new equipment. Among them, percutaneous transcatheter closure is the most popular one, but it has some disadvantages: 1) the requirement of the use of contrast agents which causes the radiation exposure to both doctors and patients; 2) non continuously real-time monitoring; 3) unclear imaging of heart structure; and 4) the dependence of patients’ age and weight as well as the size of the occluder and the methods of operation. Therefore, the application of the TEE reaches a new stage of the development of transcatheter closure technique.

TEE is developed on the basis of conventional echocardiography^[3,6]^. The probe is placed in the esophagus, which is close to the posterior wall of the left atrium without interference of the chest wall and lung gas. The advantages of TEE are: 1) clear imaging of heart structure and peripheral vessel; 2) real-time observation of the position of guiding steel wire, sheath and occluder in the heart and blood vessel; 3) clear demonstration of the position between the occluder and defect; 4) immediate observation of the effect of the occluder in case the timely adjustment is required; 5) clear display of closure of atrial or ventricular shunt and complications after closure with color Doppler ultrasound.

This study showed the decreases in the operation time, postoperative ICU stay, postoperative hospital stay, use of blood products, hospital costs and the length of incision in the TEE group compared to CPB group. In the TEE group, 13 (92.86%) patients were transferred to the general ward after 24h and 13 (92.86%) were discharged within 7 days after operation. TEE guided with a minimally invasive transthoracic occlusion technique can reduce the surgical trauma, affect less in breathing and cough, make the postoperative nursing care simple, reduce the volume of blood products transfusion. Patients in the TEE group can be transferred to ordinary ward after a short period of time of operation within, therefore reducing the total hospitalization expenses and improving the patients’ satisfaction degree on the incision. Also, the method used in pediatric patients can not be restricted by the age and weight. Another advantage for the use of TEE is that patients can be readily changed the traditional surgery once defect is not suitable for closure by using TEE or plugging failure occurs, therefore avoiding the risk and ensuring the safety.

In the present study, the success rate of occlusion in the TEE group was 100%. In our single center, the success rate of percutaneous closure of ASD surgery was about 98%. The reasons for the failure are that the defect is too large and close to the valve, causing the abnormal valve opening and closing by the occluder or that the ASD edge is too thin, impairing the occluder. However, there have been some reports showing that success rate for TEE guided ASD occlusion was 96%^[8]^. The causes of failure in ASD occlusion were relatively large defect, short residual edge or porous defects.

The limitations of this study were: 1) No control of conventional percutaneous transcatheter closure was included; 2) The comparison with CPB is not a randomized controlled study; 3) It theoretically described the advantages of simple TEE guided transthoracic minimally invasive transcatheter closure of CHD; and 4) Postoperative follow-up time is short and long-term effect needs to be have a multicenter investigation with large sample researches.

## CONCLUSION

In summary, esophageal ultrasound guided with minimally invasive transthoracic transcatheter closure for CHD is safe and feasible. It can reduce the trauma happened in the conventional open surgery. It has small incision and avoids the multiple organ injury caused by CPB, which is beneficial for the early rehabilitation of patients. In addition, it also has advantages of less drainage quantity, less blood transfusion, less postoperative complications, short hospital stay, relatively low cost in hospitalization and good social and economic benefits. However, not all ASD patients are suitable for TEE technique and it is necessary to strictly control the indications. The operation specification of TEE technique needs to have a multicenter investigation with large sample researches.

**Table:** 

Authors’ roles & responsibilities	
ZG	Conception and study design, realization of operations; analysis and/or data interpretation; statistical analysis; manuscript redaction or critical review of its content; final manuscript approval
CZ	Conception and study design; realization of operations; analysis and/or data interpretation; statistical analysis; manuscript redaction or critical review of its content; final manuscript approval
HW	conception and study design; realization of operations; analysis and/or data interpretation; statistical analysis; manuscript redaction or critical review of its content; final manuscript approval
SG	Conception and study design; realization of operations; analysis and/or data interpretation; statistical analysis; manuscript redaction or critical review of its content; final manuscript approval
